# Biogeography of *Argylia* D. Don (Bignoniaceae): Diversification, Andean Uplift and Niche Conservatism

**DOI:** 10.3389/fpls.2021.724057

**Published:** 2021-10-19

**Authors:** Nataly S. Glade-Vargas, Carla Rojas, Paola Jara-Arancio, Paula Vidal, Mary T. Kalin Arroyo, Luis Felipe Hinojosa

**Affiliations:** ^1^Departamento de Ciencias Ecológicas, Facultad de Ciencias, Universidad de Chile, Santiago, Chile; ^2^Instituto de Ecología y Biodiversidad, Santiago, Chile; ^3^Departamento de Ciencias Biológicas, Departamento de Ecología y Biodiversidad, Facultad de Ciencias de la Vida, Universidad Andrés Bello, Santiago, Chile

**Keywords:** Andes, diversity, phylogeny, niche conservatism, Bignoniaceae

## Abstract

Andean uplift and the concomitant formation of the Diagonal Arid of South America is expected to have promoted species diversification through range expansions into this novel environment. We evaluate the evolution of *Argylia*, a genus belonging to the Bignoniaceae family whose oldest fossil record dates back to 49.4 Ma. Today, *Argylia* is distributed along the Andean Cordillera, from 15°S to 38.5°S and from sea level up to 4,000 m.a.s.l. We ask whether *Argylia*’s current distribution is a result of a range expansion along the Andes Cordillera (biological corridor) modulated by climatic niche conservatism, considering the timing of Andean uplift (30 Ma – 5 Ma). To answer this question, we reconstructed the phylogenetic relationships of *Argylia* species, estimated divergence times, estimated the realized climatic niche of the genus, reconstructed the ancestral climatic niche, evaluated its evolution, and finally, performed an ancestral range reconstruction. We found strong evidence for climatic niche conservatism for moisture variables, and an absence of niche conservatism for most of the temperature variables considered. Exceptions were temperature seasonality and winter temperature. Results imply that *Argylia* had the capacity to adapt to extreme temperature conditions associated with the Andean uplift and the new climatic corridor produced by uplift. Ancestral range reconstruction for the genus showed that *Argylia* first diversified in a region where subtropical conditions were already established, and that later episodes of diversification were coeval with the of Andean uplift. We detected a second climatic corridor along the coastal range of Chile-Peru, the coastal lomas, which allowed a northward range expansion of *Argylia* into the hyperarid Atacama Desert. Dating suggests the current distribution and diversity of *Argylia* would have been reached during the Late Neogene and Pleistocene.

## Introduction

Southern South America exhibits a wide range of climates that results in an extraordinary diversity of biomes including forest, grassland, steppe, alpine, and desert (Ortega et al., 2012). The current distribution of these biomes reflects the interaction of climatic factors and historical processes at different time scales. These include past climate change, plate tectonics and mountain building.

**FIGURE 1:**
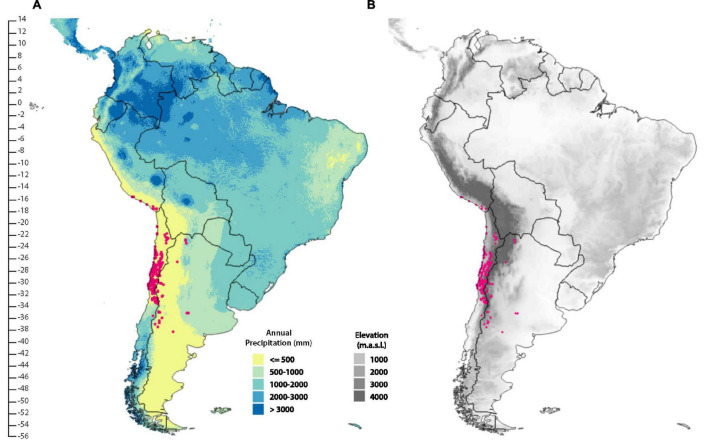
**(A)** Distribution of the genus *Argylia* in relation to the South American Arid Diagonal (SAD) (shown in yellow) and elevation **(B)**. The SAD is defined by less than or equal to 500 mm annual precipitation. The red circles are occurrences for all species in the genus.

One of the most defining features of South America biogeography is a large arid region with annual rainfall below 500 mm that crosses the western side of the continent diagonally from northwest to southeast, beginning at coastal equatorial latitudes (4°S) and culminating on the Atlantic coast at latitude 55°S. This region has been defined as the South American Arid Diagonal (SAD, Veit and Garleff, 1995; Villagrán and Hinojosa, 1997; Garreaud et al., 2010; Figure 1). SAD developed gradually during the Oligocene and Miocene as a consequence of the Andean uplift and the establishment of the current equatorial-polar temperature gradient (Hinojosa and Villagrán, 1997; Zachos et al., 2001; Dunai et al., 2005; Hinojosa, 2005). Arid conditions have been postulated for latitudes affected by the anticyclonic influence of the South America Subtropical High (24°S) in the absence of the Andes since the Late Jurassic (150 Ma, Hartley et al., 2005; Garreaud et al., 2010). The final uplift of the Andes during the Miocene defined the current pattern of rainfall distribution in the area through the rain shadow effect of the Andes blocking moist air brought by winds from the east in its northern extreme and conversely, from the west in its southern extreme. The coeval emergence of the Humboldt Current during the Neogene further intensified aridity leading to the development of the current hyperarid Atacama Desert on the Pacific coast, a region considered one of the most arid in the world (Hinojosa and Villagrán, 1997; Latorre et al., 1997; Hartley et al., 2005; Garreaud et al., 2010).

The Central Andes (4°S–46°S, Ramos, 1999) orogeny is considered to have occurred in different uplift pulses since ca. 30 Ma. The region between 14°S and 16°S reached its current elevation around ca. 5 Ma, Garzione et al. (2017) with the development of a rain shadow blocking off easterly flow today to around 27°S. In contrast, in the southernmost part (from 39°S south), uplift produced a rain shadow effect on the westerlies at 16 Ma (Ramos and Ghiglione, 2008). At a continental scale, the development of SAD led to the disjunction of the forest biome in South America, with several tree/shrub genera distributed today in South American tropical and temperate forests: *Azara*, *Blepharocalyx*, *Crinodendron*, *Escallonia*, *Myrteola*, and *Myrceugenia* (Villagrán and Hinojosa, 1997).

However, the development of SAD associated with Andean uplift would have also promoted diversification of taxa adapted to arid conditions and low temperatures, configuring the development of an arid/semi-arid biogeographic corridor. This corridor would have favored northward expansion and the evolution of Andean-Patagonian taxa. Recognition of a biogeographic corridor necessarily implies that expansion following the SAD would occur when lineages tend to retain their climatic niche requirements (Eldredge et al., 2005; Kozak and Wiens, 2007; Donoghue, 2008; Little et al., 2010; Wiens et al., 2010; Hinojosa et al., 2016). Therefore, when new environments appear, the latter would be occupied by lineages that possess the relevant adaptations for these new conditions and thus manage to colonize these areas by tracking their original climatic niches.

**FIGURE 2:**
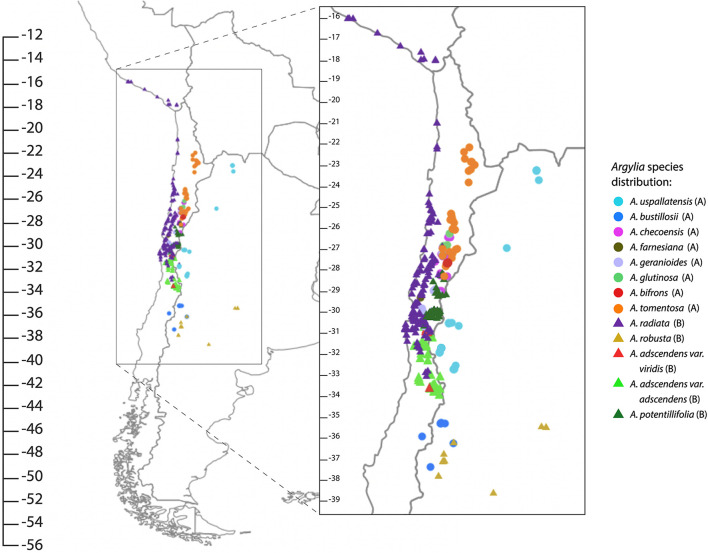
Distribution of species of *Argylia*. A and B indicate the clade to which a species belongs.

The large family Bignoniaceae Juss. comprises trees, shrubs and vines that are distributed largely in tropical areas and to a lesser extent in temperate zones (Gleisner and Ricardi, 1969; Gentry, 1980; Olmstead, 2013). The oldest fossil record attributed to Bignoniaceae dates to 49.4 Ma (Pigg and Wehr, 2002). Molecular analyses subdivide the family into nine clades made up of 82 genera and 860 species (Lohmann, 2006). The genus *Argylia* D. Don belongs to the Tecomeae clade (Olmstead, 2013) which together with the Tourrettieae and Jacarandeae clades are the earliest branching clades of the family (Olmstead et al., 2009). *Argylia* is exclusively distributed from southern Peru (coastal lomas) to the Central Andes of Chile and Argentina. Following the Arid Diagonal of South America, *Argylia* occurs along the Andes ranges, on the western and eastern slopes but mostly on the western slopes, from 15°S to 38.5°S, from sea level to 4,000 m.a.s.l. Species commonly are found above 1,200 m.a.s.l. A notable exception is *Argylia radiata* which is distributed mainly along the Pacific coast (Figure 2).

**FIGURE 3:**
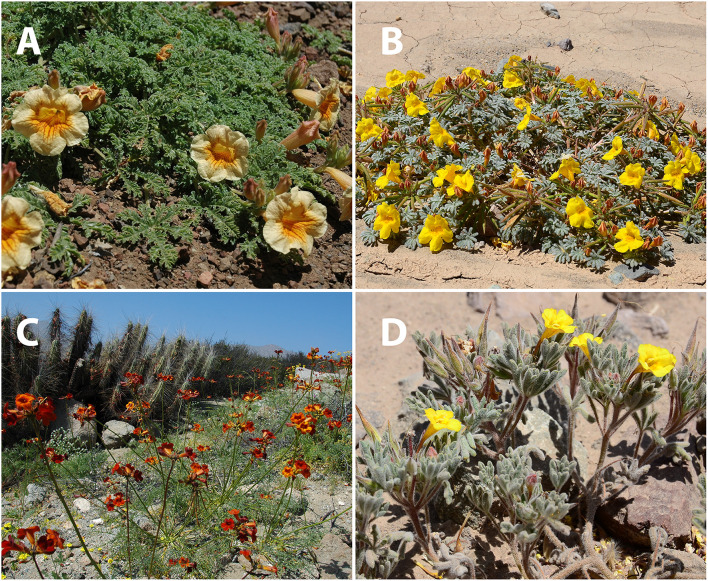
Images of four species of *Argylia* showing the range of habits found in the genus. **(A)** *A. adscendens van adscendens.* **(B)** *A. checoensis.* **(C)** *A. radiata.* **(D)** *A. tomentosa.* Image credits: Maria Teresa Eyzaguirre, Fundación R. A. Philippi.

*Argylia* is unusual in the Bignoniaceae for its perennial herb to subshrub habit and presence of thick woody roots. The palmate leaves with a long petiole are alternate to subopposite. The leaf arrangement also distinguishes *Argylia* from other genera of Bignoniaceae (Figure 3; Gentry, 1992). The large bell-shaped flowers are cream to brick-red (including within the same species). They appear adapted to bee-pollination. The genus has wind-dispersed seeds.

*Argylia* is an excellent model for understanding the role of the Andean uplift as promoter of arid environments on plant evolution, and for evaluating the hypothesis of climatic niche conservation in these new environments. In this study we propose: *Argylia* initially diversified in an area subjected to the South Pacific Subtropical High, the ancestral niche was arid, and the lineage is characterized by niche conservatism in moisture variables. This implies that the current distribution of *Argylia* was attained over the period of Andean uplift and consequently would have been affected by it. Specifically, we: (1) we establish the phylogenetic relationships of species of the genus *Argylia*, (2) determine the climatic conditions under which the genus originated, (3) evaluate climatic niche conservatism during its diversification, and finally, (4) evaluate the Andean uplift on the diversification of the genus.

## Methodology

### Taxon Sampling

DNA for *Argylia* and other species used in this study was obtained from leaf material of individuals collected in the field and from herbarium material stored CONC (Herbarium of the Department of Botany, University of Concepción), SGO (Herbarium of the National Museum of Natural History), and HULS (Herbarium of de University of La Serena). We obtained DNA of the 13 taxa of the genus *Argylia*: 10 taxa endemic to Chile [*Argylia adscendens* DC. var. *adscendens*; *A. adscendens* DC. var. *viridis* (Phil.) Gleisner & Ricardi *adscendens*; *Argylia bifrons* Phil.; *Argylia checoensis* (Meyen) I.M. Johnst.; *Argylia farnesiana* Gleisner & Ricardi; *Argylia geranioides* DC.; *Argylia glutinosa* Phil.; *Argylia potentillifolia* DC.; *A. radiata* (L.) D. Don, and *Argylia tomentosa* Phil.], two species which also occur in Argentina (*Argylia bustillosii* Phil.; *Argylia uspallatensis* DC.), and one which is endemic to Argentina (*Argylia robusta* Sandwith) (Table 1; Gleisner and Ricardi, 1969; Zuloaga et al., 2008). We incorporated several representatives of the family Bignoniaceae as an outgroup; *Tecoma capensis* (Thunb.) Lindl., *Campsidium valdivianum* (Phil.) Skottsb., four taxa of the genus *Fridericia* Mart. and one member of the Verbenaceae [*Glandularia laciniata* (L.) Schnack & Covas.]. A representative of Verbenaceae was included given that the Verbenaceae is the plant family closest to the Bignoniaceae (Stevens et al., 2001; Table 2). We used more than one individual per taxon initially to verify the positions of *Argylia* species in the topology.

**TABLE 1:** Primers used to amplify and sequence rDNA and cpDNA.

Primer	Sequence (5′-3′)	References
ITS4	TCCTCCGCTTATTGATATGC	White et al., 1990
ITS5	GGAAGTAAAAGTCGTAACAAGG	White et al., 1990
*trn*L	CGAAATCGGTAGACGCTACG	Taberlet et al., 1991
*trn*F	ATTTGAACTGGTGACACGAG	Taberlet et al., 1991
*rpL*32-F	CAGTTCCAAAAAAACGTACTTC	Shaw et al., 2007
*trn*L(UAG)	CTGCTTCCTAAGAGCAGCGT	Shaw et al., 2007

**TABLE 2:** Collection localities, herbarium voucher numbers, and GenBank accession numbers of taxa.

Taxa	Localities					GenBank		
	Country	Region/Location	Lat	Long	Voucher	ITS	*rp*L32F-*trn*L	*trn*LF
***Argylia* Genus**								
*A. adscendens* var. *adscendens* DC.	Chile	Santuario de la Naturaleza Yerba Loca, RM	33.21	70.2	CONC 166983	MZ312464	MZ327968	MZ327983
*A. adscendens* var. *viridis* DC.	Chile	Ovalle, Coquimbo	30.46	70.43	CONC 103063	MZ312465	MZ327969	MZ327984
*A. bifrons* Phil.	Chile	Copiapó, Atacama	27.3	69.37	CONC 30120	–	MZ327970	–
*A. bustillosii* Phil	Argentina	Mendoza, Mendoza	35.82	70.17	CONC 30124	MZ312466	MZ327971	MZ327985
*A. checoensis* (Meyen) I.M. Johnst.	Chile	Copiapó, Atacama	27.09	69.52	CONC 107165	MZ312467	MZ327972	MZ327986
*A. farnesiana* Gleisner & Ricardi	Chile	Cuesta de Pajonales, Coquimbo	29.09	70.58	CONC 30436	MZ312468	MZ327973	MZ327987
*A. geranioides* DC.	Chile	Río Elqui, Coquimbo	29.53	71.15	CONC 103066	MZ312469	MZ327974	MZ327988
*A. glutinosa* Phil.	Chile	Quebrada Doña Ines Chica, Atacama	26.07	69.34	CONC 168434	MZ312470	MZ327975	MZ327989
*A. potentillifolia* DC.	Chile	Quebrada la Totora, Atacama	28.42	70.12	CONC 156276	MZ312471	MZ327976	MZ327990
*A. radiata* (L.) D. Don	Chile	Lomas de taltal, Antofagasta	25.27	70.26	CONC 157513	MZ312472	MZ327977	MZ327991
*A. robusta* Sandwith	Argentina	San rafael, Mendoza	36.99	69.89	CONC 30130	MZ312473	MZ327978	MZ327992
*A. tomentosa* Phil.	Chile	Conchi Viejo y San Pedro, Antofagasta	21.58	68.36	CONC 139888	MZ312474	MZ327979	MZ327993
*A. uspallatensis* DC.	Argentina	Quebrada de Ciénaga Colgada, Argentina	31.79	70.03	CONC 75331	MZ312475	MZ327980	MZ327994
**Outgroups**								
**Bignoniaceae Genera**								
*Campsidium valdivianum* (Phil.) W. Bull	Chile	Isla Esmeralda, Magallanes y la Antártica Chilena	49.06	75.3	CONC 175000	MZ312477	MZ327981	MZ327996
*Fridericia cinnamomea* (DC.) L. G. Lohmann	Brazil	Duke Reserve	2.9	59.92	Vicentini 809 (INPA, MO)	–	KP757329	–
*Fridericia erubescens* (DC.) L. G. Lohmann	Brazil	Chapada Diamantina	11.54	41.17	Lohmann 359 (MO, SPF)	–	KP757328	–
*Fridericia sp. nogueira*	Brazil	Delphinópolis	20.34	46.85	350 (SPF)	–	KP757359	–
*Fridericia speciosa* Mart.	Brazil	PE Do Rio Doce	19.66	42.53	Lombardi 2521 (BHCB, MO)	–	KC914604	–
*Tecoma capensis* (Thunb.) Lindl.	United States	Botanical Garden, UC Berkeley	37.8	122.2	UC Berkeley Botanical Garden (50-1870)	MW854072	–	–
**Verbenaceae Genera**								
*Glandularia laciniata* (L.) Schnack & Covas	Chile	Farellones	33.35	70.3	CONC 185096	MZ312478	–	MZ327997

### DNA Extraction, Amplification, and Sequencing

Genomic DNA was extracted with the DNeasy Plant Kit (Qiagen, Valencia, CA, United States). We amplified the DNA using the primers listed in Table 1. PCR used a final volume of 30 μl, which contained 4 μl DNA (25 ng/μl), 8.35 μl distilled water, 3 μl MgCl_2_ (25 mM), 6 μl buffer, 2.4 μl of dNTP (1 mM), 1.8 μl of each primer (10x ITS4-ITS5 y *trn*LF), 2.4 μl BSA (25 mM), and 0.25 μl GoTaq (5 U/μl). DNA was denatured at 95°C for 5 min, followed by 35 amplification cycles of 45 s at 94°C, annealing for 1 min at 52°C, elongation for 1.5 min at 72°C and a final extension of 7 min at 72°C. In the case of *rpL*32-F-trnL, the thermal cycler protocol was modified: DNA was denatured at 80°C for 5 min, followed by 35 amplification cycles of 30 s at 94°C, annealing for 1 min at 56°C, elongation temperature change of 0.3° per second to the elongation temperature for 5 min at 69°C and a final extension of 7 min at 72°C. Samples were sent to Macrogen (Seoul, South Korea) for purification and sequencing. Sequences were loaded, edited and aligned using ChromasPro 2.33 (Technelysium, Brisbane, QLD, Australia) and BioEdit 7.0 (Hall, 1999). All new sequences have been deposited in GenBank (Table 2).

### Phylogenetic Analysis and Estimation of Divergence Times

We performed a combined rDNA and cpDNA analysis with a total of 3,149 nuclear characters (913 ITS, 1099 *trn*F-*trn*L, and 1495 *rpL*32-F-*trn*L, Table 2). Phylogenetic reconstruction was performed with Bayesian inference in the MrBayes version 3.2 program (Ronquist et al., 2012). Three partitions were used for each gene; the evolutionary models for each were: GTR + G in ITS; GTR + I + G in *trn*L-*trn*F; and GTR + I in *rpL*32-F-*trn*L, which were obtained with the MrModeltest program version 2.2 (Nylander, 2004). Runs appeared stationary prior to 20^6^ generations, and we conservatively excluded the first 2.0 × 10^6^ generations of each run as burn-in. The effective sample size (ESS) value was greater than 200 in a range between 1,174,899 and 1,623,107. Nodes with >0.95 were considered to be supported for posterior probabilities (Ronquist and Huelsenbeck, 2003).

Divergence times were estimated assuming a relaxed molecular clock with evolutionary models for each partition in the Beast program (version 1.4.8; Drummond and Rambaut, 2007). We used the estimated age of the genus *Fridericia*, sister clade of *Argylia*, 30.9 Ma (Lohmann et al., 2013) as a calibration point.

### Realized Climatic Niche of Extant *Argylia* Species

We used 371 occurrences for 13 specimens of *Argylia* obtained from herbarium species stored at CONC (Universidad de Concepción) and from records in the Global Biodiversity Information Facility (GBIF). These occurrences were verified with each species distribution reported in the bibliography (Gentry, 1992; Rodriguez et al., 2018) eliminating doubtful georeferences that did not correspond to what was established or, that corresponded to the location of the herbarium and not to the area where species were sampled. From the CHELSA data base, we obtained 19 bioclimatic variables with approximately 1 km^2^ resolution (Karger et al., 2017). For niche modeling, we performed a principal component analysis on the 19 bioclimatic variables selecting 11 which retained 78% of the total variance (Supplementary Figure 1): Mean Annual Temperature (MAT), Temperature Seasonality (TS), max. Temperature of the Warmest Month (maxTWaM), min. Temperature of the Coldest Month, mean Temperature of the Warmest Quarter (minTCQ), mean Temperature of the Coldest Quarter (mTCQ), Annual Precipitation (AP), Precipitation of the Driest Month (PDM), Precipitation Seasonality (PS), Precipitation of the wettest Quarter (PWeQ), and Precipitation of the Driest Quarter (PDQ).

The climatic niche for each species was modeled using the maximum entropy algorithm Maxent (Phillips et al., 2006). We used a total of 50 replicates, 25% of the data as a training set and, in order to avoid model overfitting, we defined a species-specific setting selected for Maxent using the “ENMeval” (Muscarella et al., 2014) R package. The approach implemented in ENMeval runs successively several MAXENT models using different combinations of parameters to select the settings that optimize the trade-off between goodness of fit and overfitting. We selected the parameters with the lowest AIC values (Supplementary Table 1).

### Climatic Niche Evolution

The predicted niche occupancy (PNO) profiles were used to calculate the maximum likelihood estimate and 95% confidence intervals (95% CI) for each climate variable at each interior node of the phylogeny, assuming Brownian motion evolution (Evans et al., 2009). To obtain PNO profiles with respect to the bioclimatic variables selected, we used the raw probability (RP) distribution of each species derived from Maxent. Confidence intervals were calculated using an unbiased estimate of the variance of the Brownian motion. Analyses were conducted using the R packages Phyloclim (Heibl, 2011), Ape (Paradis et al., 2004), and Phytools (Revell, 2012).

To evaluate the evolution of the climatic niche in *Argylia*, we tested phylogenetic signal estimating Pagel’s lambda (Pagel, 1994), which ranges from zero to one. Lambda (λ) is a scaling parameter for correlations between the phylogenetic similarity matrix and the trait matrix. λ = 0 signifies that trait correlations between species are independent of phylogeny. Conversely, λ = 1 indicates similarity between species equal to the Brownian motion model of evolution expectation in which case trait evolution is strongly influenced by phylogeny (Pagel, 1999). The λ parameter was estimated for each bioclimatic variable using the *phylosig* function from the R-package Phytools (Revell, 2012). To evaluate the fit of climatic niche we used the Akaike Information Criterion for three evolutionary models: (a) a Brownian motion (BM) model of gradual and continuous drift, (b) an Ornstein-Uhlenbeck (OU) model which can be thought of as a stabilizing selection model of evolution with one optimum, and (c) a White Noise (WN) model of random variation, in which climatic niche variation is independent of phylogenetic relationships (Hansen et al., 2008; Hawkins et al., 2014). Fits to these alternative models was made using the *fitContinuous* function from the R package Geiger (Harmon et al., 2008). All the analyses were conducted with R v. 3.3.3 (R Core Team, 2021).

To infer the climatic history of *Argylia*, we used the projection of our phylogenetic tree in environmental (each bioclimatic variable) and temporal space assuming Brownian motion evolution (BM; Schluter et al., 1997; Evans et al., 2009), using the R package Phytools (Revell, 2012).

### Ancestral Range Distribution Reconstruction

We inferred ancestral distributions across the *Argylia* phylogeny by comparing models that considered anagenetic evolutionary process (i.e., dispersal, extinction), with cladogenetic process (i.e., sympatry and vicariance). BioGeoBEARS R package (Matzke, 2014; R Core Team, 2021) implements widely used models of range evolution (e.g., Ree and Smith, 2008), but it includes an additional parameter of cladogenetic speciation mediated by founder events: the jump parameter “j.” This parameter allows daughter species to instantaneously “jump” outside the geographical range of parental species by long distance dispersal or dispersalism. A likelihood ratio test was performed to reject the null hypothesis that the incorporation of the parameter “J” in each model confer equal likelihoods on data. Finally, the Akaike Information Criterion (AIC) was used to compare the relative fit of the six models and choose the best model of ancestral range reconstruction (Matzke, 2014). The models compared were: Dispersal extinction cladogenesis (DEC) and DEC + J; a likelihood dispersal-vicariance analysis (DIVAlike) and DIVALIKE + J and Bayesian biogeographical inference model (BAYAREALIKE) and BAYAREALIKE + J, a likelihood version where no particular cladogenetic events occurred. Our analysis considered four areas for ancestral range reconstruction, taking into account the altitudinal and latitudinal characteristics of the present distribution of *Argylia* species: Pacific Coast (A), Central Andes between 20°S and 33°S (B), Central Andes south 33°S, southern limit of Central Andes flat slab (C), Patagonia (D). These areas were defined according to a geological definition for Central Andes (Ramos, 1999) and according to current distribution of the genus. This region was divided into north of 33°S and south of 33°S. The latter corresponds to the Central Andes Flat Slab region, segment where there is an absence of volcanic activity (Charrier et al., 2007). Central Andes, the main area of our study, also corresponds to the Ecoregion of Central Andes according to Olson et al. (2001). On the other hand, South of 33°S is an area where precipitation occurs during winter and is associated to the Southern Andean Steppe Ecoregion (Olson et al., 2001). Finally, we defined the area “Pacific coast” to represents the distribution of the Atacama-Sechura desert Ecoregion (Olson et al., 2001). And finally, we defined the “Patagonia” area, to describe the distribution along the Patagonian Pampa Ecoregion (Olson et al., 2001).

We constrained our analysis to the Andean uplift. For this we divided our multiplier matrix (Supplementary Figure 2) into four time periods: (1) 50-35 My defined by the global cooling event; (2) 35-15 My defined by the glaciation of east Antarctica; (3) 15-5 MY defined by Andean uplift prior to modern elevation; 5–0 My defined by the culmination of Andean uplift.

Finally, we estimated the number and type of biogeographical events that account for the present distribution of *Argylia.* For this we used biogeographical stochastic mapping (BSM) implemented in “BioGeoBEARS” (Matzke, 2015).

## Results

### Phylogenetic Analysis and Divergence Times

**FIGURE 4:**
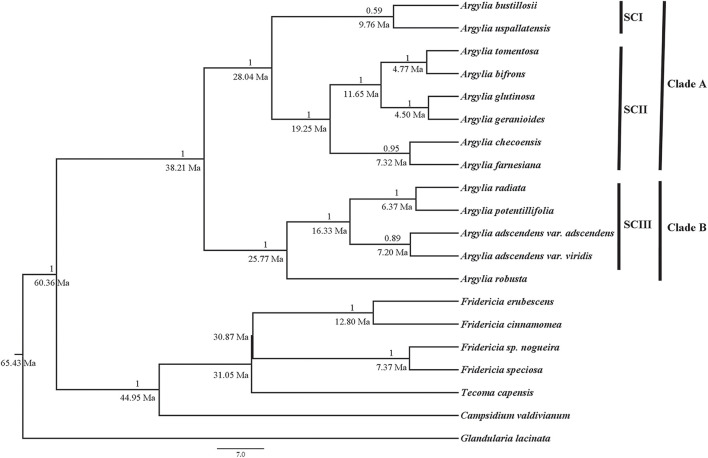
Bayesian inference phylogenetic tree based on combined analysis (rDNA and cpDNA) for 13 taxa of the genus *Argylia* and seven taxa belong to the Bignoniaceae and Verbenaceae. Posterior probability values are found above the branches. Estimation of divergence times are found below the branches. The principal lineages are indicated by clade A and clade B, and subclades SCI, SCII, and SCIII.

The results show that *Argylia* is a monophyletic group with an estimated minimum date of origin of ∼38.21 Ma (Figure 4). It is composed of two main clades and four subclades. Clade A comprises eight taxa. It diverged ∼28.04 Ma to further subdivide into two subclades: SC I, formed by the two Chilean-Argentinean taxa *A. uspallatensis* and *A. bustillosii*, with a minimum age of ∼9.76 Ma, and SC II, composed of six Chilean taxa: *A. tomentosa*, *A. bifrons, A. glutinosa, A. geranioides*, *A. checoensis, A. farnesiana*, with a minimum age of ∼19.25 Ma. Clade B, with a minimum age of ∼25.77 Ma, comprises five taxa. This clade is separated into *A. robusta*, which occurs exclusively in Argentina (Mendoza, Neuquén, and Río Negro) is sister to SC III subclade, which is formed by the Chilean species *A. potentillifolia, A. radiata*, *A. adscendens* var. *adscendens* and *A. adscendens* var. *viridis*, with a minimum age of ∼16.33 Ma.

### Realized Climatic Niche and Niche Evolution

**FIGURE 5:**
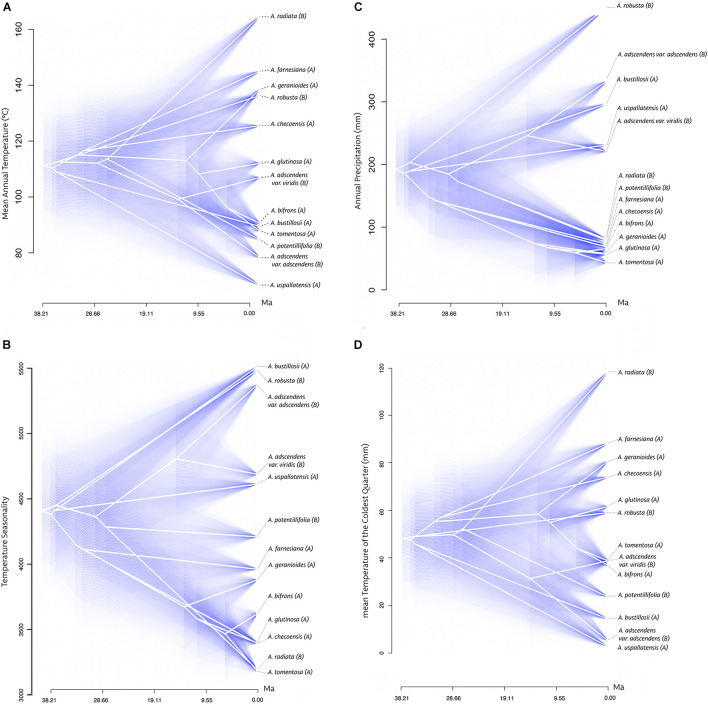
Traitgram of ancestral states of *Argylia* climatic niche. White lines correspond to a projection of the phylogenetic tree in a space defined by each bioclimatic variable. **(A)** Mean annual temperature (°C); **(B)** Temperature Seasonality; **(C)** Annual Precipitation (mm); **(D)** mean Temperature of de Coldest Quarter (°C). Blue shade areas correspond to the 95% of confidence interval. Dashed lines to phylogenetic tree tips link the names of *Argylia* species. Clades of species are shown in parenthesis: (A) and (B) as in Figure 4.

Climatic niche models obtained with Maxent performed consistently well. The average training AUC for 50-replicate runs were all above 0.9 (Table 3). Extant species of *Argylia* are primarily found in microthermal climatic conditions with a Mean Annual Temperature ranging from 7°C to 13.4°C and an Annual Precipitation ranging from 20 to 477 mm (Figure 5 and Table 3). Exceptionally, *A. radiata* and *A. farnesiana* are found in marginally mesothermal conditions, as shown by Mean Annual Temperature above 14°C.

**TABLE 3:** Bioclimatic variables for species of the genus *Argylia*, along with the corresponding phylogenetic clade and subclade (see Figure 4).

Clade	Subclade	Species	MAT	TS	maxTWaM	minTCM	mTWaQ	mTCQ	AP	PDM	PS	PWeQ	PDQ	AUC
A	I	*A. bustillosii*	8.95	550.98	22.47	−3.14	16.46	1.44	296.82	9.92	46.43	138.73	34.37	1.00
		*A. uspallatensis*	6.85	461.27	18.93	−5.12	12.84	0.29	231.12	4.25	76.38	136.33	15.67	0.99
	II	*A. bifrons*	9.07	362.02	19.67	−2.44	13.63	3.71	65.73	1.80	107.59	38.14	6.48	1.00
		*A. checoensis*	12.55	339.44	22.74	0.75	16.69	7.39	68.57	0.79	130.34	45.95	2.70	1.00
		*A. farnesiana*	14.5	396.2	24.7	3	19.5	8.8	73	0	129	65	0	–
		*A. geranioides*	13.84	388.00	24.40	1.78	18.64	8.04	57.76	0.01	120.07	45.15	1.98	1.00
		*A. glutinosa*	11.20	339.74	21.16	0.28	15.51	6.16	56.69	1.69	102.23	29.73	6.18	1.00
		*A. tomentosa*	8.80	318.30	19.20	−3.22	12.64	3.89	43.43	0.57	131.25	31.36	1.92	1.00
B	III	*A. potentillifolia*	8.48	421.00	19.68	−3.58	13.77	2.37	78.80	0.28	128.18	65.66	1.10	1.00
		*A. radiata*	16.45	319.33	25.34	6.38	20.50	11.80	80.74	0.21	122.80	62.40	1.25	0.99
		*A. robusta*	13.63	548.34	26.32	1.09	21.03	5.90	450.47	13.53	47.25	193.85	45.57	0.97
		*A. ads. var. viridis*	10.70	468.84	22.41	−1.98	16.63	3.88	220.93	0.53	102.68	152.15	5.45	1.00
		*A. ads. var. adscendens*	7.83	537.23	20.92	−4.73	14.92	0.55	332.44	0.97	105.72	237.26	6.21	1.00

*Values are the weighted mean of each bioclimatic variable derived from predicted niche occupancy. AUC, area under the curve; MAT, Mean Annual Temperature; TS, Temperature Seasonality; maxTWaM, maximum Temperature of the Warmest Month; minTCM, minimum Temperature of the Coldest Month; mTWaQ, mean Temperature of the Warmest Quarter; mTCQ, mean Temperature of the Coldest Quarter; AP, Annual Precipitation; PDM, Precipitation of the Driest Month; PS, Precipitation Seasonality; PWeQ, Precipitation of the Wettest Quarter; PDQ, Precipitation of the Driest Quarter.*

Traitgrams associated with precipitation in *Argylia* show that sister species are more similar to one another than to their more distant relatives, suggesting phylogenetic niche conservatism of these variables (Figure 5C and Supplementary Figures 3A–D). On the contrary, bioclimatic variables associated with temperature generally show no phylogenetic niche conservatism. In this case, species grouping along the temperature axes show multiple crossing between the two clades (Figure 5A and Supplementary Figures 3E–G). Exceptionally, traitgrams for Temperature Seasonality and winter temperature (mean Temperature of the Coldest Quarter) shows niche conservatism (Figures 5B,D).

Pagel’s phylogenetic signal test shows the same pattern. Precipitation variables presented strong phylogenetic signal, indicated by λ significantly different from zero and equal to 1 (*p* ≤ 0.05, Table 4). The rest of the temperature bioclimatic variables fit better to the null model of evolution (White Noise), suggesting phylogenetic independence in the evolutionary history of these traits. Exceptionally, Temperature Seasonality (Bio4) and mean Temperature of the Coldest Quarter (Bio11), shows phylogenetic niche conservatism. Winter temperature (mean Temperature of the Coldest Quarter) showed marginally significant niche conservatism (*p* = 0.055, Table 4).

**TABLE 4:** Results of phylogenetic signal (Pagel’s Lambda) and Weighted Akaike, based on exp (−0.5 × ΔAIC) to compare the best fit between a Brownian Motion (BM) model, an Ornstein-Uhlenbeck (OU) model and a White Noise (WN) null model of evolution.

Bioclimatic variable	WAIC			Phylogenetic signal	
	BM	OU	WN	λ	*p*
MAT	0.35	0.107	0.543	7.77E-05	1
TS	0.768	0.136	0.096	1.055	0.039
maxTWaM	0.355	0.13	0.515	7.77E-05	1
minTCM	0.375	0.103	0.523	7.77E-05	1
mTWaQ	0.351	0.132	0.517	7.77E-05	1
mTCQ	0.462	0.094	0.444	1.174	0.055
AP	0.807	0.143	0.051	1.158	0.006
PDM	0.776	0.137	0.087	1.134	0.018
PS	0.662	0.117	0.221	0.96	0.135
PWeQ	0.815	0.144	0.041	1.169	0.004
PDQ	0.775	0.137	0.088	1.126	0.02

*MAT, Mean Annual Temperature; TS, Temperature Seasonality; maxTWaM, maximum Temperature of the Warmest Month; minTCM, minimum Temperature of the Coldest Month; mTWaQ, mean Temperature of the Warmest Quarter; mTCQ, mean Temperature of the Coldest Quarter; AP, Annual Precipitation; PDM, Precipitation of the Driest Month; PS, Precipitation Seasonality; PWeQ, Precipitation of the Wettest Quarter; PDQ, Precipitation of the Driest Quarter.*

### Ancestral Range Distribution Reconstruction

**FIGURE 6:**
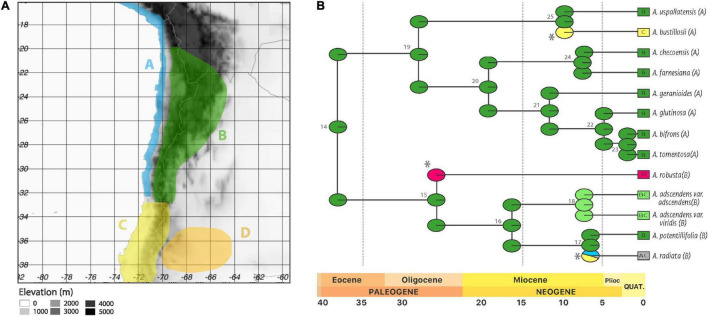
Results for the Ancestral Range Reconstruction. **(A)** Range distribution of the four areas defined by the ancestral range reconstruction A: Pacific Coast, B: Central Andes 20°S–33°S, C: Central Andes 33°S south and D: Patagonia. **(B)** Maximum likelihood ancestral range estimation, using the best model BAYAREALIKE+j. The pie diagrams at nodes show the relative probability of the possible states (areas or combinations of areas). The boxes on the right show the native ranges of taxa within these clades, as in **(A)**. The asterisks mark founder events and the small numbers in gray indicate node numbers.

Ancestral range estimations under the best fit model (BAYAREALIKE + j, Table 5) showed that the most probable ancestral area for extant species of *Argylia* is the Central Andes (B, Figure 6). A summary of Biogeographical Stochastic Mappings (BSMs) revealed that most biogeographical events comprise within-area speciation (64.2%) and cladogenetic dispersal (21.4%), with few areal expansions (14.4%) (Table 6 and Supplementary Figure 4).

**TABLE 5:** Comparative statistical analyses to compare the best fit between biogeographical models: DEC, dispersal-extinction-cladogenetic model; DIVALIKE, likelihood dispersal-vicariance model; BAYAREALIKE: Bayesian biogeographical inference model.

Alternative model	Null model	LnL		Chi-squared	AIC		AICw	
		Alt. model	Null model	*p*	Alt. model	Null model	Alt. model	Null model
DEC + J	DEC	−20.73	−19.55	1	47.45	43.1	0.1	0.9
DIVALIKE + J	DIVALIKE	−20.11	−20.61	0.32	46.22	45.22	0.38	0.62
BAYAREALIKE + J	BAYAREALIKE	−19.32	−23.41	0.0043	44.64	50.81	0.96	0.044

*“j” parameter incorporates the occurrence of founder event in each model. Chi-square test was performed to compare between nested models (alternative versus null).*

**TABLE 6:** Summary of biogeographical stochastic mapping counts for *Argylia* using the BAYAREALIKE + j model.

Mode	Type	Mean (SD)	%
Within area speciation	Speciation	9 (0)	62.2
	speciation – subset	0	0
Dispersal	Founder event	3 (0)	21.4
	Range expansion	2.02 (0.14)	14.4
	Range contraction	0	0
Vicariance	Vicariance	0	0
Total		14 (0.14)	100

*Mean numbers of the different types of events estimated are shown here along with standard deviations.*

The inclusion of the “j” parameter consistently improved model fit, suggesting that range expansions alone are not sufficient to account for movement into new areas (Table 5). Three founder events were identified. The first was defined by dispersal from the Central Andes 20°S to 33°S (B) to Patagonia (D), indicated in red (Figure 6). The second founder event developed from Central Andes 20°S to 33°S (B) to Central Andes 33°S south (C), indicated with yellow in *A. bustillosii* (Figure 6). A range expansion was detected, from Central Andes 22°S to 33°S to Central Andes 33°S south, defining the current distribution of *A. adscendens* clade (BC, Figure 6), between Middle and Upper Miocene. The last founder event involved the Central Andes 22°S–33°S (B) to the Central Andes 33°S south (C) with later range expansion toward the Pacific Coast (A), defining the current distribution of *A. radiata* (indicated in gray in Figure 6).

## Discussion

### Phylogeny and Climatic Niche Evolution

According to Lohmann et al. (2013), the Bignoniaceae node where *Argylia* occurs in the family Bignoniaceae has a date of origin of ∼49.8 Ma, while Olmstead et al. (2009) indicated that *Argylia* is one of the earliest-branching clades of the family. This agrees with the ages we obtained in this study, which show a minimum origin time for *Argylia* of ∼38.2 Ma (Late Eocene). Two main clades are distinguished in the *Argylia* phylogeny (Figure 4). Clade A diversified during the Oligocene at ∼28.0 Ma, while clade B diversified around 25.8 Ma.

Extant species of *Argylia* are distributed mainly on microthermal (11°C in average) and arid conditions (below 500 mm of annual precipitation), as shown by our climatic niche model, apart from *A. radiata* which inhabits mesothermal conditions (16°C, Table 3). This species has a very large latitudinal distribution (15°S–33°S).

Lack of phylogenetic signal in most of the temperature variables indicates that evolution in *Argylia* is mediated by temperature adaptation associated. The broad current altitudinal distribution of the genus, where most of *Argylia* species are distributed between 2,000 and 4,500 m.a.s.l., reflects adaptation to progressively lower temperatures over steep elevational gradients. Exceptionally, Temperature Seasonality had a strong phylogenetic signal (λ = 1, Table 4), indicating that throughout its evolutionary history, *Argylia* species tracked annual contrasting climatic conditions. Winter temperature (mean Temperature of the Coldest Quarter), which strongly determine temperature variation (or seasonality), also showed phylogenetic signal, although it was only marginally significant (see Table 4).

Ancestral reconstruction for temperature seasonality shows that *Argylia* must have been distributed in regions with a significant thermal amplitude (Supplementary Table 2). This condition is associated with continentally, where annual temperature fluctuation increases from coastal to inland areas (Driscoll and Fong, 1992). The current distribution of *Argylia* seems to correlate broadly with this pattern (Supplementary Figure 5), reinforcing the idea that *Argylia’s* evolution was driven by the emergence of the Arid Diagonal, as we hypothesized.

The requirement of seasonality condition for *Argylia’s* climatic niche can also be observed with precipitation variables that presented a strong phylogenetic signal, such as winter and summer precipitation (Precipitation Seasonality, Precipitation of the Driest Month, Precipitation of the Wettest Quarter and Precipitation of the Driest Quarter, Figure 5, Table 4, and Supplementary Figure 3). Even though, climatic seasonality is not the only determinant requirement for the genus, but also the availability of moisture seems to have a strong effect over *Argylia’s* evolution and diversification, given by the strong phylogenetic conservatism of Annual Precipitation (Figure 5A and Table 4).

These results are especially important under current climate change scenarios. Especially in northern and central Chile, where there is a 30% of precipitation deficit and a historic increase in temperatures (Center for Climate and Resilience Research [CR2], 2015). Considering the projection of a decrease in precipitation for the 2100 year (Qin et al., 2014), high Andean genera with strong precipitation conservation, such as *Argylia*, could be at risk. Indeed, a reduction in precipitation is likely to have much more severe consequences that an increase in temperature.

### Ancestral Range Reconstruction

The timing of Andean uplift and late Cenozoic global climate change can be observed in the biogeographical reconstruction of *Argylia* (Figure 6 and Ramos and Ghiglione, 2008; Giambiagi et al., 2016; Garzione et al., 2017). Our result suggests that the Central Andes between 20°S and 33°S would be the area were *Argylia* initially diversified. Three founder effects were found in our biogeographical model (Table 6, Figure 6, and Supplementary Figure 4).

**FIGURE 7:**
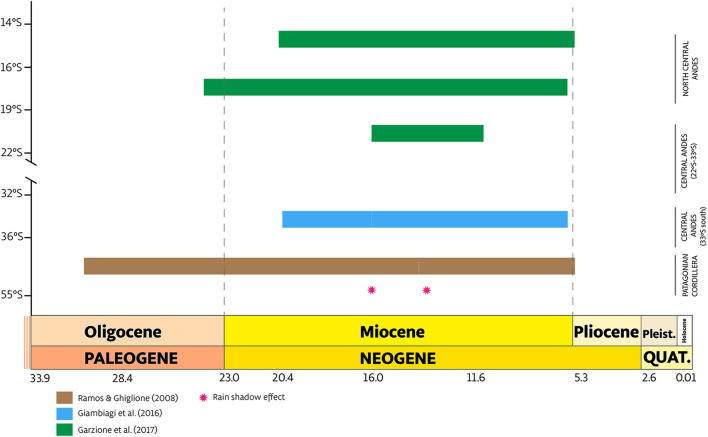
Schematic diagram of the time of uplift along the Northern Central Andes, Central Andes (20°S–33°S and 33°S south) and Patagonian Andes based on Ramos and Ghiglione (2008), Garzione et al. (2017), and Giambiagi et al. (2016). The geological timeline is shown in Ma. Red stars provide an estimation of when the rain shadow associated with elevation came into force.

The first, allowed the colonization of the Extra Andean area, located south of 32°S at 25.77 Ma. This limit has been previously recognized as a distributional boundary for some *Argylia* species (Gentry, 1992) and has also been recognized as the southern part of the Central Andean Flat Slab (Ramos and Folguera, 2009). During the Oligocene, both isotopic and fossil evidence show a drop of temperature and precipitation (Zachos et al., 2001; Hinojosa, 2005), coeval with the uplift of Patagonian Andes (Ramos and Ghiglione, 2008; Figure 7), suggesting semi-arid conditions on Patagonia by the time of this colonization. Aridity would also be reinforced by the effect of the anticyclonic subsidence and global temperature decline, which in turned increased equator-pole climatic gradient (Zachos et al., 2001; Hartley et al., 2005).

The last two founder events occurred independently in the evolutionary history of *Argylia*, resulting in colonization of Central Andes 33°S south from the ancestral area (Central Andes 22°S–33°S, Figure 6). These dispersal events occurred between 15 and 5 Ma, by which time the Andean uplift pulses had already begun (Figures 6, 7; Ramos and Ghiglione, 2008; Giambiagi et al., 2016; Garzione et al., 2017). This period corresponds to the modern climate in South America stemming from the establishment of the Antarctic circumpolar current, the modern atmospheric circulation pattern and reinforcement of the Humboldt Current in the Pacific Ocean (Hinojosa and Villagrán, 1997; Hartley et al., 2005; Garreaud et al., 2010). These events are a sign of the birth of the Andean corridor, allowing north-south dispersal of species (Luebert and Weigend, 2014). This corridor has been considered as an explanation for plant diversification patterns in South America, such as in: *Azorella*, *Chuquiraga*; *Laretia*, *Leucheria*, *Mulinum*, and *Perezia* (Ezcurra, 2002; Simpson et al., 2009; Nicolas and Plunkett, 2012, 2014; Pérez et al., 2020). Apparently, this holds true also for *Argylia*, where dispersal and cladogenetic events occurred mainly during a period when arid and semi-arid altitudinal belts were formed. However, formal climatic niche analyses, as performed here, are lacking for the above-mentioned genera.

Two range expansion were detected within B clade (Figure 6). The first expansion occurred from Central Andes 22°S to 33°S (ancestral area, B) to Central Andes 33°S south (C), between Middle and Upper Miocene during the Andes Mountain building (Figure 7; Giambiagi et al., 2016), allowing a climatic corridor between both areas and range expansion of *A. adscendens* Clade (*var. adscendens* and *var. viridis*). The second expansion occurred from Central Andes 33°S south to Pacific Coast (A), which allowed *A. radiata* to colonize the coastal area as far north as southern Peru (Figure 2; Gentry, 1992; Rodriguez et al., 2018). *Argylia*’s northward expansion (Figure 6) suggest a second corridor along the Pacific Coast at the end of Cenozoic and during the Quaternary as suggested by its distribution. Today, this species is distributed from 33°S to 15.8°S, occupying the coastal range along the subtropical West of South America (Figure 2), where moisture comes mainly from the interception of low coastal clouds, a phenomenon known as the “Camanchaca.” Coastal topography (Rundel et al., 1991; Garreaud et al., 2008) associated with the Camanchaca has allowed the development of high species richness in the coastal Atacama and Sechura Deserts comprising the Lomas Formation (Hueck and Seibert, 1972; Rundel et al., 1991). The main drivers of the Camanchaca are the strong air subsidence associated with the South American Subtropical High (SASH), the annual variability of SASH, the constant temperature inversion by the cool Humboldt Current and the uplift of the coastal topography (Rundel et al., 1991; Garreaud et al., 2008, 2010). The age of the Humboldt Current and elevated coastal topography dates to Middle/Upper Neogene and Pleistocene, respectively (Hinojosa and Villagrán, 1997; Villagrán et al., 2004; Hartley et al., 2005; Garreaud et al., 2010; Regard et al., 2021). These ages are consistent with our ancestral range reconstruction and the strong phylogenetic signal on moisture variables found in this study. It would be worthwhile testing this phylogeographic hypothesis at the population level over the entire distributional range *A. radiata*.

All the above expansions were mediated by the last pulse of Andean uplift (5 Ma, Figures 6, 7), as well by the emergence of hyper arid conditions along with the birth of the Atacama Desert (Villagrán and Hinojosa, 1997; Hartley et al., 2005), as clearly shown by the current pattern of the northernmost distribution of the genus, which presents an east-west disjunction among the species distributed in this region.

Diversification and development of novel adaptations promoted by arid conditions have been widely established in other South American plant genera: *Chaetanthera*, *Malesherbia*, *Cristaria*, *Heliotropium* sect. *cochranea, Leucheria*, and *Leucocoryne* (Gengler-Nowak, 2002; Guerrero et al., 2013; Jara-Arancio et al., 2014, 2017; Böhnert et al., 2019; Pérez et al., 2020), in accordance with the idea that arid regions are strong drivers of lineage diversification (Stebbins, 1952).

According to Luebert and Weigend (2014), high elevation taxa showed divergence times that correspond mainly with Andean uplift in the late Miocene and early Pliocene, with some taxa also diversifying in Quaternary times. The authors also highlight a north to south trend for high-Andean lineages, with lineages with more recent origin in the north and older lineages in the south. Additionally, in Chile from 18°S to 35°S, phylogenetic diversity increases from north to south, following a precipitation gradient. Both patterns could be explained by the presence of a younger arid environment that favored the diversification in this new biome (Scherson et al., 2017; Böhnert et al., 2019).

Finally, the proposition of range expansion of *A. radiata* through a corridor from Central Andes south 33°S to Pacific Coast up to 15°S in Peru, by the Late Miocene (Figure 6), would be associated with what Guerrero et al. (2013) described as a widespread temporal lag between the establishment of arid and hyperarid climates and a later diversification through Atacama-Sechura Desert, recorded in genera *Nolana, Chaetanthera*, and *Malesherbia*. This temporary lag may be a consequence of the later establishment of the second climatic corridor that we propose in this study, the coastal lomas, between the Upper Miocene and early Quaternary (Regard et al., 2021).

## Conclusion

In this work we evaluated the role of the Andean uplift and consequently the development of the Arid Diagonal in the evolution of the *Argylia* lineage. We postulated that: (a) an early diversification of *Argylia* in an ancestral area under arid conditions (given by the presence of the high-pressure system of the South Pacific Subtropical High), (b) Phylogenetic niche conservatism of climatic requirements, and (c) dispersal and later diversification of *Argylia* modulated by the Andean uplift and its role as a biological corridor.

That all precipitations variables considered were highly conserved indicates that the distribution of *Argylia* is strongly determined by moisture availability. Our results also showed that *Argylia’s* climatic niche is determined by annual temperature fluctuations, indicating that the ancestral range of *Argylia* must have been in a region where the Andes was uplifting.

The striking difference between temperature and precipitation variables is a novel finding and one that should be looked for in other Andean genera. Is this a general tendency, or variable among taxa? If it is a general condition, it requires an intrinsic physiological explanation.

On the other hand, the ancestral area of distribution for the genus was north of 32°S along the Andean range. At the time of the origin of *Argylia*, this would have been a region where subtropical conditions were already present. Later dispersal and diversifications occurred coeval with Andean uplift acting as a “corridor.” A second climatic corridor is postulated by our analysis, where the northward expansion into the hyperarid zone in the coast is associate with an extra moisture supply by the marine low clouds Camanchaca. Thus, *Argylia* tracked its moisture requirements as suggested by the strong phylogenetic signal in these environmental variables. At the end of the Neogene and during the Pleistocene, the *Argylia* lineage would have reached its modern distribution and associated species diversity.

Finally, *Argylia’s* biogeographic history highlights the value of niche conservatism studies where the availability of climatic corridors and moisture environmental filters are essential for species range expansions in the context of modern climate change.

## Data Availability Statement

The original contributions presented in the study are publicly available. This data can be found here: https://www.ncbi.nlm.nih.gov/genbank/. Accession number(s) can be found in the article/Supplementary Material.

## Author Contributions

NG-V, MA, LH, PJ-A, and CR: conception and design of the study. NG-V, PJ-A, PV, and CR: acquisition of the data. NG-V, LH, and PJ-A: analysis and/or interpretation of the data. NG-V, LH, and MA: drafting and revising the manuscript. All authors contributed to the article and approved the submitted version.

## Conflict of Interest

The authors declare that the research was conducted in the absence of any commercial or financial relationships that could be construed as a potential conflict of interest.

## Publisher’s Note

All claims expressed in this article are solely those of the authors and do not necessarily represent those of their affiliated organizations, or those of the publisher, the editors and the reviewers. Any product that may be evaluated in this article, or claim that may be made by its manufacturer, is not guaranteed or endorsed by the publisher.
