# Supplementary material for: Biogeography of Argylia D. Don (Bignoniaceae): Diversification, Andean Uplift and Niche Conservatism
Source: Front Plant Sci. 2021 Oct 19;12:724057. doi: 10.3389/fpls.2021.724057 (PMC8579820; doi:10.3389/fpls.2021.724057)
Supplement: Supplementary file 4 [file Data_Sheet_1.PDF]

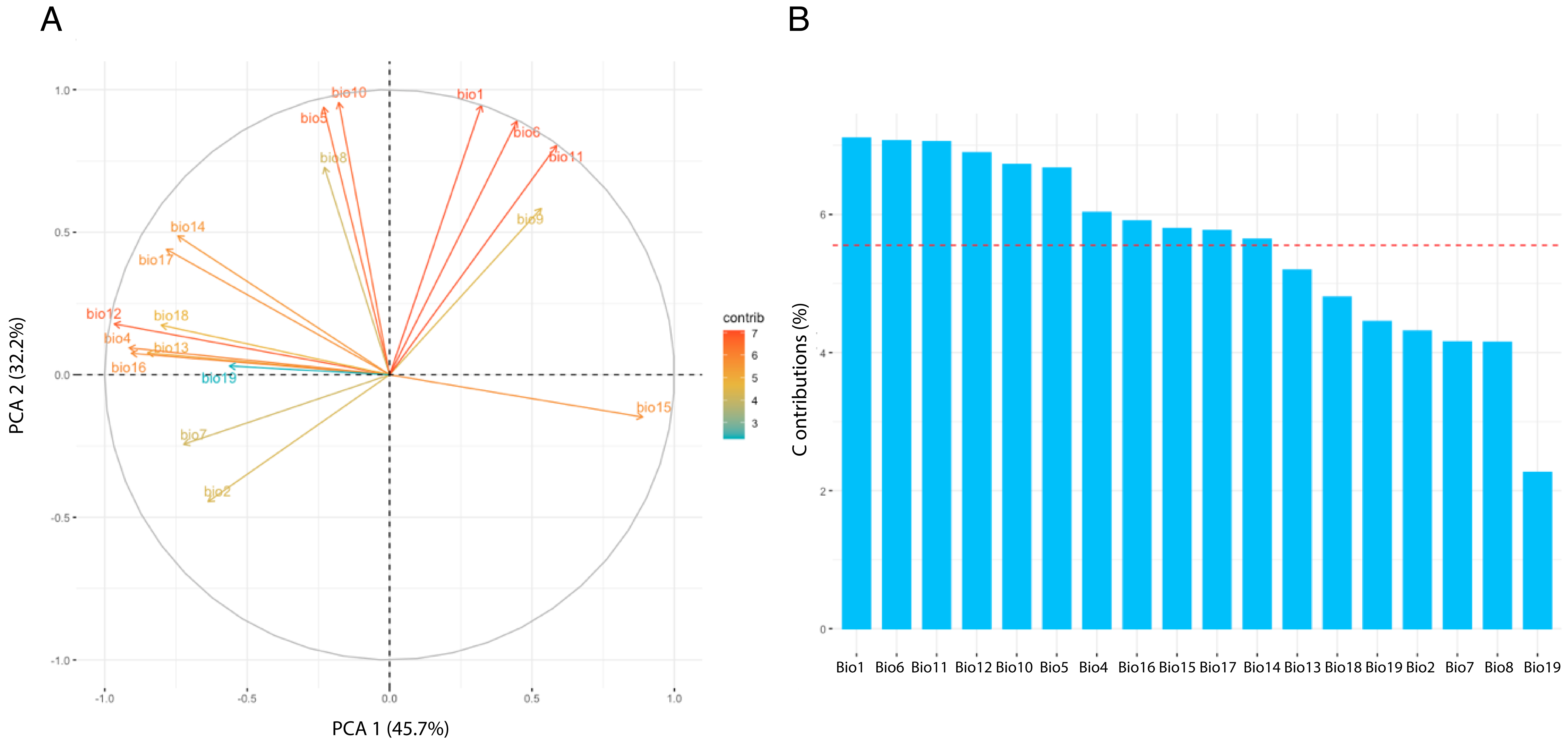


**Supplementary Figure 1**. Results of principal component analysis for 19 bioclimatic variables. A. Biplot for the first two principal components (78% variance). B. Plot of the contribution of each bioclimatic variable. The doted red line shows the expected value if contributions were uniform. Bio 1: Mean Annual Temperature. Bio 4: Temperature Seasonality. Bio 5: max. Temperature of the Warmest Month. Bio 6: min. Temperature of the Coldest Month. Bio 10: mean Temperature of the Warmest Quarter. Bio 11: mean Temperature of the Coldest Quarter. Bio 12: Annual Precipitation. Bio 14: Precipitation of the Driest Month. Bio 15: Precipitation Seasonality. Bio 16: Precipitation of the Wettest Quarter. Bio 17: Precipitation of the Driest Quarter.
