# Supplementary material for: Biogeography of Argylia D. Don (Bignoniaceae): Diversification, Andean Uplift and Niche Conservatism
Source: Front Plant Sci. 2021 Oct 19;12:724057. doi: 10.3389/fpls.2021.724057 (PMC8579820; doi:10.3389/fpls.2021.724057)
Supplement: Supplementary file 5 [file Data_Sheet_2.PDF]

5 Ma

	NCst.	NAnd.	CAnd	EAnd.
NCst.	1	1	1	0
NAnd.	1	1	1	0
CAnd.	1	1	1	1
EAnd.	0	0	1	1

15 Ma

	NCst.	NAnd.	CAnd	EAnd.
NCst.	1	0	0.5	0
NAnd.	0	1	0.5	0
CAnd.	0.5	0.5	1	0.5
EAnd.	0	0	0.5	1

35 Ma

	NCst.	NAnd.	CAnd	EAnd.
NCst.	1	0	0	0
NAnd.	0	1	0.3	0
CAnd.	0	0.3	1	0.3
EAnd.	0	0	0.3	1

50 Ma

	NCst.	NAnd.	CAnd	EAnd.
NCst.	1	0	0	0
NAnd.	0	1	0	0
CAnd.	0	0	1	0
EAnd.	0	0	0	1

Supplementaty Figure 2. Data table for a time-stratified analyses for ancestral range distribution reconstruction of the genus Argylia in 5 time periods: 5, 15, 35 and 50 million of years ago. NCst: North Coast; NAnd.: North Andes; CAnds.: Centrl Andes; EAnd.: Extra Andes, as in Figure 5.
