# Supplementary material for: Biogeography of Argylia D. Don (Bignoniaceae): Diversification, Andean Uplift and Niche Conservatism
Source: Front Plant Sci. 2021 Oct 19;12:724057. doi: 10.3389/fpls.2021.724057 (PMC8579820; doi:10.3389/fpls.2021.724057)
Supplement: Supplementary file 7 [file Data_Sheet_4.PDF]

A. Range switching dispersal "a" event counts

	NCst.	NAnd.	CAnd	EAnd.
NCst.	-	0	0	0
NAnd.	0	-	0	0
CAnd.	0	0	-	0
EAnd.	0	0	0	-

C. Cladogenetic dispersal event counts "j" (Standard deviations)

	NCst.	NAnd.	CAnd	EAnd.
NCst.	-	0	0.6 (0.49)	0
NAnd.	0.6 (0.49)	-	1.4 (0.49)	1 (0)
CAnd.	0	0	-	0
EAnd.	0	0	0	-

B. Range expansion "d" event counts (Standard deviations)

	NCst.	NAnd.	CAnd	EAnd.
NCst.	-	0	0.6 (0.49)	0
NAnd.	0	-	1.02 (0.14)	0
CAnd.	0.4 (0.49)	0	-	0
EAnd.	0	0	0	-

D. All dispersal event counts "a+d+j" (Standard deviations)

	NCst.	NAnd.	CAnd	EAnd.
NCst.	-	0	0.6 (0.49)	0
NAnd.	0.6 (0.49)	-	2.42 (0.54)	1 (0)
CAnd.	0.4 (0.49)	0	-	0
EAnd.	0	0	0	-

Supplementary Figure 4. Number of dispersal events estimated in the history of Argylia with biogeographical stochastic mapping. Counts of dispersal events were average across 50 BSMs and are represented with standard deviation in parentheses. Anagenic dispersal events are shown in A and B. Total cladogenetic dispersal events are shown in C. These counts are mean values of 50 BSMs. Standard deviations are shown in parenthesis. NCst: North Coast; NAnd: North Andes; CAnd: Central Andes; EAnd: Extra Andes.
