# Supplementary material for: Biogeography of Argylia D. Don (Bignoniaceae): Diversification, Andean Uplift and Niche Conservatism
Source: Front Plant Sci. 2021 Oct 19;12:724057. doi: 10.3389/fpls.2021.724057 (PMC8579820; doi:10.3389/fpls.2021.724057)
Supplement: Supplementary file 8 [file Data_Sheet_5.PDF]

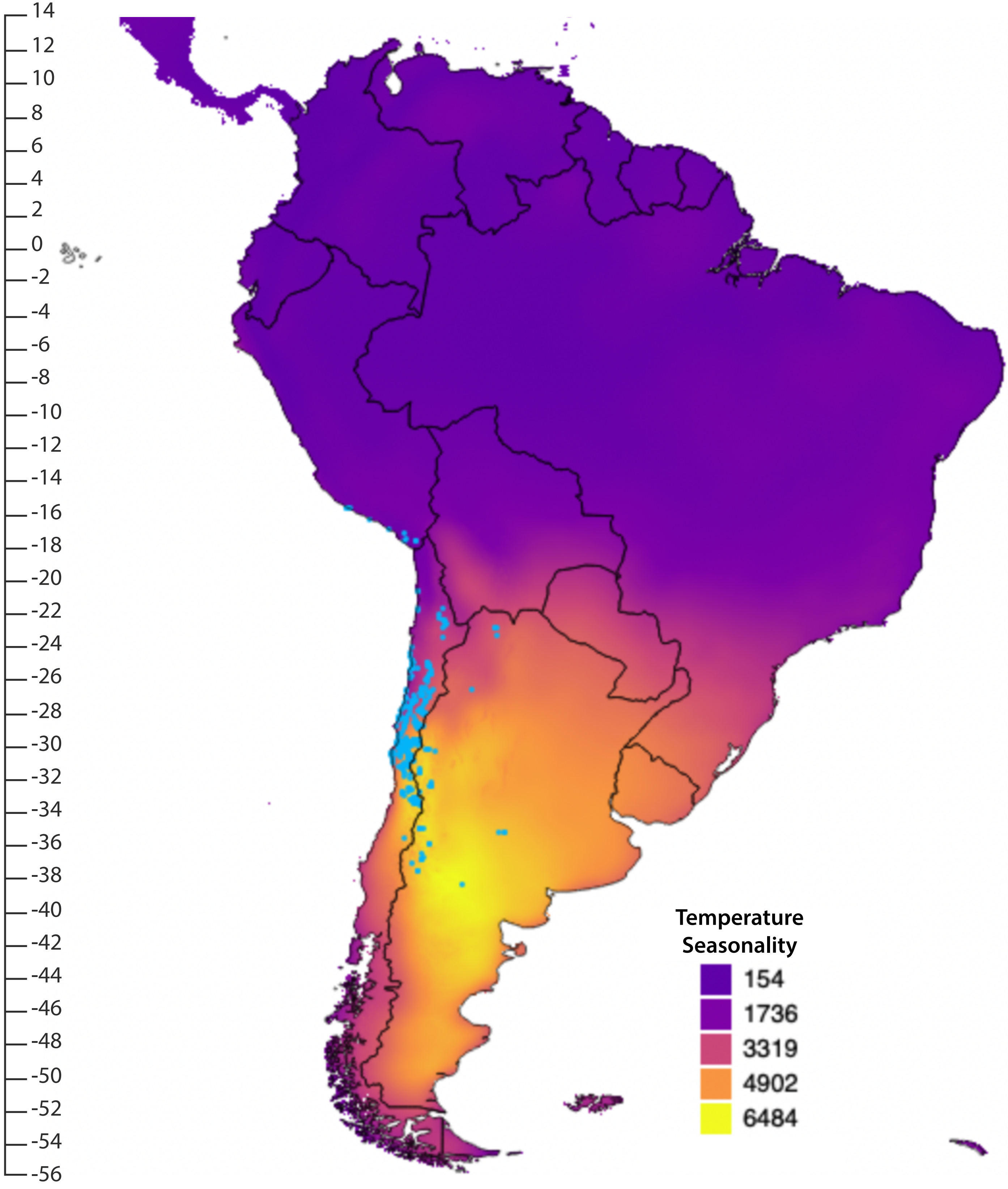

Supplementary Figure 5. Distribution of species of *Argylia* (in light blue) along a Temperature Seasonality gradient (Bio 4).
